# Utilizing Quinoa Flour for Functional Lavash Bread Production

**DOI:** 10.1002/fsn3.70533

**Published:** 2025-07-04

**Authors:** Mustafa Akturfan, Suzan Yalçın

**Affiliations:** ^1^ Department of Gastronomy and Kitchen Arts, Faculty of Applied Sciences Karamanoğlu Mehmetbey University Karaman Turkey; ^2^ Department of Food Hygiene and Technology, Faculty of Veterinary Medicine Selçuk University Konya Turkey

**Keywords:** bread fortification, functional food, nutritional value, rheological parameters, wheat lavash bread

## Abstract

Lavash bread is a traditional food in West Asia and the Middle East, with its consumption increasing in recent years. This study aimed to assess the impact of adding 5%, 10%, 15%, and 20% quinoa flour on the rheological properties of wheat dough, as well as the sensory and chemical characteristics of lavash bread. As the quinoa flour content increased, sedimentation value and mixing tolerance index decreased, while water absorption, dough development time, and dough softening increased. The addition of quinoa flour reduced the extensograph energy value and extensibility but increased the extensograph ratio of elasticity to extensibility. Lavash bread made with quinoa flour remained light in appearance, though its lightness (L*) decreased, and redness (a*) and yellowness (b*) increased with higher quinoa content. Quinoa also improved the chewiness and flexibility of the lavash bread without affecting its appearance, taste, or odor. Notably, the overall acceptability of the bread increased as the quinoa flour content rose. From a nutritional perspective, protein, lipid, fiber, sugar, and ash contents increased, while starch content decreased with the addition of quinoa. Furthermore, significant increases in the levels of aspartic acid, alanine, arginine, valine, leucine, and lysine were observed. In conclusion, incorporating 20% quinoa flour into lavash bread enhances its nutritional value and functional properties, making it a healthier alternative to traditional wheat lavash.

## Graphical abstract

This study investigated the effects of incorporating 5%–20% quinoa flour into lavash bread. The addition of quinoa improved dough rheology, enhanced protein, fiber, and mineral content, and maintained sensory acceptability. Lavash enriched with 15%–20% quinoa flour showed optimal nutritional and functional qualities.

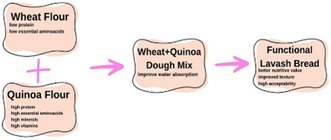

## 1 Introduction

The bakery industry is one of the fastest‐growing sectors in the food industry. Flatbreads have been a staple food for centuries with over 60 varieties produced worldwide. Lavash bread, one of the most popular flatbreads in Turkey, is traditionally made from wheat flour. However, wheat flour contains limited amounts of essential amino acids, particularly lysine, tryptophan and threonine, and lacks the amino acid balance necessary for optimal biological utilization, as noted by Dhingra and Jood (2002) and Aghamirzaei et al. (2013). Furthermore, wheat‐based breads are typically low in vitamins A, C, D, and B_12_. The incorporation of pseudo‐cereal flours such as quinoa into wheat‐based products offers a promising approach to enhancing their nutritional quality. These additions can increase protein content, improve the amino acid profile, and enrich the final product with dietary fiber, vitamins, and minerals (Codina et al. 2016).

In recent years, various plant‐based flours, including corn (Hussein et al. 2013), chickpea (Man et al. 2015), pumpkin (Wongsagonsup et al. 2015), lupine (Ahmed 2013), quinoa (Codina et al. 2016), and amaranth (Berghofer and Schönlechner 2000) have been used in composite flour formulations to produce functional foods. The alternative flours influence the physical, chemical, and nutritional properties of baked goods, directly impacting human health (Codina et al. 2016; Noorfarahzilah et al. 2014).

Quinoa ( *Chenopodium quinoa* Willd.) is a pseudo‐cereal belonging to the Amaranthaceae family and traditionally referred to by the Incas as the “mother of all grains,” has attracted increasing scientific interest due to its high nutritional value (Hirose et al. 2010; El‐Sohaimy et al. 2019). It contains a well‐balanced composition of amino acids, including significant amounts of lysine and methionine. Its protein content typically ranges between 12% and 18%, exceeding that of conventional cereals such as wheat and corn. In addition to its high protein quality, quinoa is rich in dietary fiber, minerals, particularly phosphorus, magnesium, calcium, iron, and essential vitamins such as vitamin E and B group vitamins and antioxidants (Codina et al. 2016; Hirose et al. 2010; El‐Sohaimy et al. 2019).

According to the study of Turkut et al. (2016) 25% quinoa flour can successfully be incorporated into commercial gluten‐free bread formulations without creating any negative effect on sensory properties. Enriquez et al. (2003) reported that replacing 10% of wheat flour with quinoa flour improved protein content while maintaining desirable loaf volume. Codina et al. (2016) found that incorporating 5%–10% quinoa flour enhanced bread quality by increasing its nutrient content and improving sensory attributes. Similarly, Stikic et al. (2012) noted positive effects on the nutritional, rheological, and sensorial characteristics of dough with the inclusion of 20% dehulled quinoa flour.

Understanding how these alternative flours affect dough characteristics requires detailed rheological analysis. The Brabender Farinograph is widely used to measure dough properties such as water absorption, stability, softening degree, and development time (Khan et al. 2021). In addition, the Extensograph provides important information regarding dough resistance and extensibility by measuring parameters such as maximum resistance to extension (Rmax, elasticity), resistance at 5 cm extension (R5), extensibility (E), the viscoelastic ratio (extensograph ratio, Rmax/E), and extensograph energy (AACC 2000).

Although there are considerable researches on the use of quinoa flour in some bakery products (Enriquez et al. 2003; Codina et al. 2016; Turkut et al. 2016; Kurek and Sokolova 2019; Ma et al. 2022), limited studies have focused specifically on its application in lavash bread. Therefore, this study aims to investigate the impact of different levels of quinoa flour dough on wheat flour dough rheology, lavash bread quality, including color and sensory properties, and its nutritional composition.

## 2 Materials and Methods

Quinoa seeds were purchased from the commercial supplier and then ground into flour. Lavash bread was produced by adding quinoa seed flour to normal bread wheat flour at the levels of 0%, 5%, 10%, 15%, and 20% in a commercial bakery in Konya‐Turkey. In lavash bread production, bread wheat flour, quinoa seed flour, water, fresh yeast, and salt were used. Each group production was carried out in six replicates.

### 2.1 Chemical Composition

The analysis of moisture (AOAC method 930.15, 2000), crude protein (AOAC method 990.03, 2000), ether extract (AOAC method 920.39, 2000), crude fiber (AOAC method 978.10, 2000), crude ash (AOAC method 942.05, 2000), sugar (AOAC method 925.35, 2000), and starch (AOAC method 996.11, 2000) in quinoa flour, wheat flour, and lavash bread was performed according to the methods of AOAC (2000). The levels of calcium, phosphorus, potassium, magnesium, iron, copper, zinc, and manganese were determined using ICP‐MS (Agilent 7500‐ce model, Yamanashi‐Ken, Japan).

Amino acid analysis was done by the HPLC (Agilent 1100 serial, Agilent Technologies, Waldbronn, Germany) using modified OPA derivatization. Gluten levels were also determined in wheat flour and quinoa flour (AACC 2000). Sedimentation analysis of flour samples was also done according to the method of AACC (2000).

### 2.2 Rheological Properties of Dough Samples

To determine the rheological properties of dough samples formulated with wheat control and its blends with quinoa wholemeal, physicochemical analyses were conducted using farinograph and extensograph tests.

In the farinograph test, the farinograph test device (Brabender Farinographe, Germany) was used with AACC method 54–21.02 (AACC 2000) for the measurement of flour water absorption (WA, %), dough development time (min, time to reach maximum consistency), dough stability (min, time during dough consistency is at 500 Brabender Units, BU), mixing tolerance index (BU), and degree of softening after 12 min.

Extensographic properties were determined using Extensograph test device (Brabender Extensographe, Germany) with AACC method 54‐10.01 (AACC 2000). Taking into account the 135‐min bread‐making process, the dough's extensibility, resistance to extension, maximum resistance to extension, and dough energy (extensograph energy) were determined. Additionally, the extensograph ratio was calculated by comparing Rmax to E.

### 2.3 Color Measurement

The color of lavash samples according to the L*, a*, and b* coordinate system was measured using Minolta Chroma meter (CR‐400, Konika Minolta, Tokyo, Japan). Color of samples were measured in L* (0 = black, 100 = white), a* (− value = green, + value = red), b* (− value = blue, + value = yellow) as reported by Rathee et al. (2024). Every measurement was done from 6 different points in lavash samples.

### 2.4 Sensory Evaluation

Sensory evaluation of lavash breads was conducted 2 h after baking by a panel of eight trained individuals who had prior experience in sensory analysis of cereal‐based products, using a five‐point hedonic scale. The panelists rinsed their mouths with tap water and swallowed water between samples. The following sensory indicators were evaluated: appearance, taste, odor, chewiness, elasticity, and overall acceptability. The score ranged from 1 (extremely dislike) to 5 (extremely like) (Esposito et al. 2024).

### 2.5 Statistical Analysis

IBM SPSS Statistics for Windows, Version 23.0. Armonk, NY, USA: IBM Corp. was used for statistical analyses. Each production group consisted of six replicates (*n* = 6). The Kolmogorov–Smirnov test was applied for the normality of data distribution. One‐way ANOVA was used to detect the effects of using different levels of quinoa flour on different parameters. Comparisons among means were done by the Tukey test. Polynomial contrasts were used to determine the linear and quadratic effects of quinoa flour on different parameters. The statistical significance level was accepted as *p* < 0.05.

## 3 Results and Discussion

The fortification of wheat bread has led to the development of new baking products with enhanced nutritional value. Quinoa is recognized as an important food source due to its high protein quality, essential amino acid content, and balanced composition of protein and lipids (Nascimento et al. 2014). The analysis of quinoa flour revealed that it is richer in protein, lipid, ash, fiber, minerals, and amino acids compared to wheat flour as shown in Table 1. In particular, quinoa flour exhibited higher levels of leucine, arginine, lysine, tyrosine, and methionine. It also contained greater amounts of essential minerals such as iron, zinc, and manganese. Owing to its high lysine content—which compensates for the deficiency of this essential amino acid in wheat flour—quinoa flour improves the nutritional quality of wheat‐quinoa composite flours. While wheat flour was found to contain approximately 29% gluten, quinoa flour was confirmed to be inherently gluten‐free in the present study, making it a suitable alternative for individuals with gluten sensitivity, as reported by Stikic et al. (2012) and Rizzello et al. (2016).

**TABLE 1:** Composition of wheat flour and quinoa flour.

Nutrients	Wheat flour	Quinoa flour
Moisture (%)	10.75	10.02
Ash (%)	0.75	2.06
Crude protein (%)	11.80	13.80
Ether extract (%)	1.78	5.80
Crude fiber (%)	0.82	2.10
Sugar (%)	3.80	3.90
Starch (%)	69.53	56.09
Gluten (%)	29	0
Minerals		
Calcium (g/kg)	0.25	2.05
Phosphorus (g/kg)	1.22	3.32
Potassium (g/kg)	3.09	8.98
Magnesium (g/kg)	0.44	1.45
Iron (mg/kg)	9.40	81.05
Copper (mg/kg)	1.93	2.28
Zinc (mg/kg)	12.2	21.37
Manganese (mg/kg)	9.8	19.73
Amino acids (%)		
Aspartic acid	0.30	1.00
Glutamic acid	2.06	1.55
Serine	0.46	0.50
Proline	1.12	0.98
Glycine	0.41	0.30
Alanine	0.49	0.80
Arginine	0.33	0.87
Threonine	0.27	0.38
Valine	0.50	0.73
Isoleucine	1.17	0.69
Leucine	0.32	1.14
Tyrosine	0.26	0.40
Phenylalanine	1.47	0.64
Lysine	0.20	0.53
Histidine	0.21	0.36
Methionine	0.18	0.29
Cysteine	0.26	0.28
Tryptophan	0.42	0.50
Cystine	0.42	0.35

Sedimentation value is widely used indicator for evaluating both the quantity and quality of gluten in flour (Pekmez 2018). In the present study, the sedimentation value was found to be 30.75 mL for wheat flour alone, and it slightly decreased to 29 mL when 5% quinoa flour was incorporated (Table 2). However, the inclusion of 10%, 15%, and 20% quinoa flour in the lavash bread dough significantly (*p* < 0.001) reduced the sedimentation value, which ranged from 24.75 to 18.25 mL. The sedimentation value decreased linearly (*p* < 0.001) as the proportion of quinoa flour in the blend increased. These findings show that the gluten quantity and quality were considerably lower in lavash breads containing 10%, 15%, and 20% quinoa flour as indicated as also noted by Pekmez (2018).

**TABLE 2:** Rheological and physical parameters of lavash doughs containing different percentages of quinoa flour.

	Quinoa flour (%)					SEM	*p* (significance)		
	0	5	10	15	20		Combined	Linear	Quadratic
Sedimentation value (mL)	30.75^a^	29.00^ab^	24.75^bc^	23.00^cd^	18.25^d^	1.145	< 0.001	< 0.001	0.489
Water absorption (%)	61.20^b^	61.25^b^	61.28^b^	61.88^a^	61.70^a^	0.069	< 0.001	< 0.001	0.656
Dough development time (min)	2.55^b^	2.18^b^	2.30^b^	5.13^a^	5.18^a^	0.321	< 0.001	< 0.001	< 0.001
Dough stability time (min)	8.28^a^	7.53^b^	6.88^c^	6.43^d^	5.58^e^	0.215	< 0.001	< 0.001	0.377
Mixing tolerance index (BU)	94.25^a^	91.50^a^	83.25^b^	81.75^b^	73.75^c^	1.784	< 0.001	< 0.001	0.519
Dough softening after 12 min (BU)	65.25^d^	78.00^c^	79.75^c^	108.50^b^	138.75^a^	6.136	< 0.001	< 0.001	< 0.001
Extensibility, E (mm)	120.75^a^	103.50^b^	94.75^c^	77.25^d^	69.25^e^	4.277	< 0.001	< 0.001	0.142
Resistance to extension (EU)	494.00^d^	579.00^b^	612.25^a^	602.50^a^	549.25^c^	9.899	< 0.001	< 0.001	< 0.001
Maximum resistance to extension, Rmax (EU)	594.25^b^	655.00^a^	635.00^a^	611.00^b^	561.50^c^	7.681	< 0.001	< 0.001	< 0.001
Extensograph energy value (cm^2^)	92.25^a^	88.50^a^	78.75^b^	59.75^c^	47.75^d^	3.951	< 0.001	< 0.001	< 0.001
Ratio of Rmax/E	4.09^d^	5.60^c^	6.47^b^	7.81^a^	7.95^a^	0.335	< 0.001	< 0.001	0.001

Abbreviation: SEM, standard error of mean.

^a,b,c,d,e^ Means within a row followed by the different superscripts differ significantly (*p* < 0.05).

The incorporation of quinoa flour significantly affected the rheological properties of the dough. As the proportion of quinoa flour increased, water absorption and dough softening (after 12 min) also increased (*p* < 0.001). These changes demonstrated a clear linear trend in response to the rising levels of quinoa flour (*p* < 0.001). The water absorption value of wheat flour alone was recorded as 61.20% in this study, which is consistent with the findings of Kurek and Sokolova (2019). The observed significant increase (*p* < 0.001) in water absorption with higher quinoa flour inclusion may be attributed to the elevated protein content in the composite doughs. The relationship is supported by Kurek and Sokolova (2019), who reported a strong correlation between protein content and water retention capacity in dough systems.

Water absorption is influenced by several components, including protein, starch, and fiber content (El‐Sohaimy et al. 2019; Li et al. 2020). Previous studies have consistently shown that increasing the proportion of quinoa flour in wheat flour‐based blends enhances water absorption capacity (Enriquez et al. 2003; El‐Sohaimy et al. 2019; Tomoskozi et al. 2011; Ma et al. 2022). This improvement is largely attributed to the presence of β‐type starch granules in quinoa, which have a greater surface area and thus a higher capacity to bind water molecules, enhancing overall dough hydration (Hong et al. 2018; Zi et al. 2019; Ma et al. 2022). Enhanced water absorption improves the uniformity and stability of the dough within the gluten‐starch matrix, ultimately contributing to better processing performance and bread quality (Adedara and Taylor 2021).

Farinograph dough development time is a critical indicator of flour protein quality (Dizlek and Özer 2017). Stronger flours generally exhibit longer dough development times than weaker ones (Meral and Doğan 2013).

The incorporation of quinoa flour significantly increased dough development time at 15% and 20% levels (*p* < 0.001). These results align with previous studies reporting that quinoa supplementation prolongs dough development time (Tomoskozi et al. 2011; El‐Sohaimy et al. 2019). This prolongation is likely due to differences in the physicochemical characteristics of wheat and quinoa flours. In particular, the higher water absorption capacity of quinoa which can be attributed to its elevated soluble protein content, may contribute to this effect (El‐Sohaimy et al. 2019). Furthermore, the starch composition of quinoa has been identified as a key factor influencing dough development time (El‐Sohaimy et al. 2019). Supporting this, Kurek and Sokolova (2019) also emphasized that quinoa flour significantly influences the mixing properties and increases water absorption, without reducing dough development time.

Dough stability, which reflects dough strength, is generally characterized by longer times, indicating a stronger gluten network (El‐Sohaimy et al. 2019). Similarly, Başar et al. (2016) emphasized that dough stability time serves as an indicator of a flour's resistance to mechanical mixing, with longer stability times being associated with higher flour strength. Several studies have reported that the incorporation of quinoa flour can increase dough stability time (Rodriguez‐Sandoval et al. 2012; El‐Sohaimy et al. 2019). However, in the present study, a significant and linear decrease in dough stability time (*p* < 0.001) was observed with the inclusion of quinoa flour at 5%, 10%, 15%, and 20% levels. This result aligns with the findings of Morita et al. (2001), who reported a reduction in dough stability when quinoa flour was added in the range of 7.5% to 20%. These contradictory findings across studies may be attributed to differences in the compositional and functional properties of the wheat and quinoa flours used, variations in processing conditions, and differences in the type of final product being evaluated. Enriquez et al. (2003) reported that substituting wheat flour with 5%–10% quinoa flour improved bread‐making quality, whereas higher substitution levels (15%) adversely affected dough development. In contrast, Stikic et al. (2012) observed no significant change in dough rheological properties when wheat flour was partially replaced with 10%–15% quinoa flour. Stikic et al. (2012) suggested that wheat gluten proteins may form new bonds with quinoa proteins, potentially stabilizing the dough structure and mitigating negative impacts on dough strength.

Regarding the mixing tolerance index, a significant decrease (*p* < 0.001) was observed in doughs containing 10%, 15%, and 20% quinoa flour. As the quinoa flour content increased, the mixing tolerance index values decreased consistently, indicating enhanced dough tolerance to mixing. This suggests that higher levels of quinoa flour may contribute to improved dough handling characteristics under mixing conditions.

The degree of softening increased significantly with the incorporation of higher levels of quinoa flour, particularly at the 20% substitution level in the present study (*p* < 0.001). In contrast, El‐Sohaimy et al. (2019) observed no significant change in softening up to 20% quinoa flour; however a marked increase was reported at 30% inclusion compared to wheat flour. The observed increase in softening is likely attributed to the dilution of the gluten network and a consequent reduction in gluten concentration. This weakening of the protein matrix compromises dough structure, rendering it more susceptible to softening and diminishing its viscoelastic properties, which are essential for dough stability (Enriquez et al. 2003; El‐Sohaimy et al. 2019).

In the present study, the inclusion of quinoa flour in lavash dough led to a significant and linear decrease (*p* < 0.001) in both Extensograph energy value and extensibility. Interestingly, elasticity was found to be higher at 5% and 10% quinoa flour substitution, and lower at 20%. Additionally, the extensograph energy values were significantly higher in blends containing 10%, 15%, and 20% quinoa flour compared to wheat flour alone (*p* < 0.001). The incorporation of quinoa flour also resulted in significant increases (*p* < 0.001) in resistance to extension, maximum resistance to extension, and the ratio of elasticity to extensibility. A strong linear correlation (*p* < 0.001) was observed between these rheological parameters and the quinoa flour concentration. These metrics are widely used to assess the quality of composite flours, identify appropriate raw materials, and evaluate their processing suitability for various bakery applications (Pekmez 2018).

The Extensograph is a valuable tool for evaluating the viscoelastic behavior of dough, where optimal dough performance is typically characterized by a balance between extensibility and resistance to extension. Elasticity is primarily determined by the gluten content in the dough (El‐Sohaimy et al. 2019). Enriquez et al. (2003) reported that adding 5%–15% quinoa flour to wheat flour reduced extensibility, due to the lack of gluten‐like proteins in quinoa. Similarly, El‐Sohaimy et al. (2019) noted only slight effects on elasticity and extensibility at lower quinoa levels, but observed that the ratio of elasticity to extensibility increased with higher quinoa inclusion. Although no significant differences were detected between the control and 5% and 10% quinoa flour blends, the highest elasticity/extensibility ratio was recorded at a 25% substitution level (El‐Sohaimy et al. 2019).

Extensibility is a critical factor in cereall chemistry, as it directly influences dough handling, cooking performance, and final product texture (Anderssen et al. 2004). According to Pekmez (2018), maximum resistance values, which are strongly associated with final product, are significantly influenced by the protein content of the flour. Although higher protein levels are generally expected to enhance both extensibility and maximum resistance values (Aydoğan et al. 2013), the current study found that increased protein content in the wheat‐quinoa blends was associated with reduced extensibility. This apparent contradiction may be explained by a limited increase in protein quantity or the distinct structural characteristics of proteins present in quinoa flour. As Meral and Doğan (2013) suggested, achieving high extensibility must be accompanied by adequate resistance to maintain gas cell stability and preserve dough structure, which are essential for desirable end‐product quality.

Color is an important parameter for assessing the quality of quinoa‐wheat flour bread. Lavash bread made exclusively from wheat flour exhibited a very light color. As the proportion of quinoa flour increased, the lightness (L*) value significantly decreased (*p* < 0.001), whereas the redness (a*) (*p* < 0.001) and yellowness (b*) (*p* = 0.049) values significantly increased (Table 3). Both linear and quadratic trends were observed between quinoa flour concentration and the L* value (*p* < 0.001). These results are consistent with the findings of some researchers (Codina et al. 2016; El‐Sohaimy et al. 2019), who also reported that the addition of quinoa flour to wheat flour led to a decrease in brightness (L*) and an increase in a* and b* values. Similarly, Kurek and Sokolova (2019) found that breads containing higher levels of quinoa flour appeared darker than bread made with wheat flour alone. Stikic et al. (2012) noted that breads enriched with 10%, 15%, and 20% quinoa flour exhibited a yellow‐reddish, crispy crust. Wang et al. (2015) also observed an increase in the darkness and redness of bread with rising quinoa flour levels. According to Ruffino et al. (2010), quinoa seeds contain natural pigments such as carotenoids and chlorophyll, which contribute to the coloration of both quinoa flour and the final baked products. Furthermore, El‐Sohaimy et al. (2019) suggested that the darker crusts associated with higher concentrations of quinoa concentrations may result not only from the naturally darker color of quinoa flour but also from elevated levels of reducing sugars and lysine‐rich proteins, which participate in non‐enzymatic Maillard reactions during baking.

**TABLE 3:** Color parameters of lavash breads containing different percentages of quinoa flour.

Color parameter	Quinoa flour (%)					SEM	*p* (significance)		
	0	5	10	15	20		Combined	Linear	Quadratic
L*	76.21^a^	70.95^bc^	72.25^b^	71.46^b^	68.80^c^	0.504	< 0.001	< 0.001	< 0.001
a*	3.09^b^	5.38^a^	5.11^a^	5.27^a^	6.87^c^	0.257	< 0.001	< 0.003	0.001
b*	27.74^b^	32.14^ab^	34.73^a^	29.76^ab^	32.35^ab^	0.815	0.049	0.049	0.082

Abbreviation: SEM, standard error of mean.

^a,b,c^ Means within a row followed by the different superscripts differ significantly (*p* < 0.05).

In the present study, no significant differences were observed in most sensory properties of lavash bread samples containing different levels of quinoa flour compared to wheat flour (Table 4). However, chewiness was significantly higher in the sample with 20% quinoa flour (*p* = 0.003), and a clear linear increase in chewiness was observed with rising quinoa flour content (*p* < 0.001). Elasticity also showed a significant and linear increase as the proportion of quinoa flour increased (*p* < 0.001). In terms of overall acceptability, breads containing 5%, 10%, and 15% quinoa flour received scores similar to the wheat control, while the 20% quinoa flour bread had the highest overall acceptability score (*p* = 0.021). A significant linear trend was observed in overall acceptability with increasing quinoa levels (*p* = 0.002), indicating a positive sensory response to higher quinoa inclusion.

**TABLE 4:** Sensory properties of lavash breads containing different percentages of quinoa flour.

Sensory properties	Quinoa flour (%)					SEM	*p* (significance)		
	0	5	10	15	20		Combined	Linear	Quadratic
Appearance	4.10	4.10	4.17	4.10	4.45	0.064	0.365	0.130	0.305
Taste	3.69	3.90	3.72	3.97	4.03	0.069	0.438	0.122	0.811
Odor	3.72	3.90	3.93	3.90	4.07	0.069	0.640	0.160	0.905
Chewiness	3.31^b^	3.86^ab^	3.86^ab^	3.86^ab^	4.24^a^	0.075	0.003	< 0.001	0.569
Elasticity	3.34^b^	3.97^a^	4.10^a^	4.03^a^	4.31^a^	0.073	< 0.001	< 0.001	0.124
Overall acceptability	3.76^b^	4.14^ab^	4.14^ab^	4.17^ab^	4.48^a^	0.068	0.021	0.002	0.853

Abbreviation: SEM, standard error of mean.

^a,b^ Means within a row followed by the different superscripts differ significantly (*p* < 0.05).

Codina et al. (2016) reported similar findings, noting that while wheat bread scored 7.94 for overall acceptability, the addition of 5% and 10% quinoa flour increased the score to 8.29 and 8.76, respectively. The texture of the bread was also influenced by quinoa flour content, as demonstrated in the study by Kurek and Sokolova (2019), where lower inclusion levels (5%–10%) increased bread hardness, while higher levels decreased it. Similarly, Codina et al. (2016) noted that quinoa addition initially increased hardness, but excessive levels led to softening. This behavior may be linked to starch gelatinization characteristics, since quinoa flour gelatinizes at 52°C compared to 55°C for wheat flour (Wolter et al. 2013). Iglesias‐Puig and Haros (2013) further explained that quinoa starch and lipids alter the dough's thermal properties, affecting the gelatinization process. Additionally, Kurek and Sokolova (2019) highlighted that fiber in whole quinoa flour helps maintain water balance within the dough matrix.

Chewiness, defined as the energy required to prepare food for swallowing (Păucean et al. 2019), was positively correlated with hardness, as chewier products remain in the mouth longer and are harder to break down (Păucean et al. 2019; Bourne 2002). Rizzello et al. (2016) found that 12.5% quinoa flour reduced elasticity and increased dryness, they also reported improvements in taste, color, and salinity. Kurek and Sokolova (2019) have noted that alternative flours may reduce breadmaking due to lower swelling capacity and consumer acceptance, quinoa flour supplementation up to 20% enhanced the sensory appeal of lavash bread. Stikic et al. (2012) also found that breads containing 10%, 15%, and 20% quinoa flour received excellent sensory scores, with a yellow‐reddish crust color and a very pleasant taste and aroma.

As presented in Table 5, the chemical composition of lavash bread was significantly influenced by the inclusion of quinoa flour. The average moisture content of the samples ranged from 31.96% to 33.82%. A gradual decrease in moisture was observed with increasing quinoa flour content. In contrast, the ash and sugar contents significantly increased in breads containing 10%, 15%, and 20% quinoa flour compared to the control made with 100% wheat flour. Overall, the incorporation of quinoa flour resulted in notable increases (*p* < 0.001) in protein, lipid, fiber, ash, sugar, and mineral contents, while starch levels decreased. These changes exhibited a strong linear relationship with increasing quinoa flour levels. These results are consistent with those reported by El‐Sohaimy et al. (2019) and Stikic et al. (2012), who found that the incorporation of quinoa flour into bread formulations significantly increased the protein, lipid, ash and fiber contents compared to control breads made solely from wheat flour.

**TABLE 5:** Proximate composition of lavash breads containing different percentages of quinoa flour (as is basis).

Nutrients (%)	Quinoa flour (%)					SEM	*p* (significance)		
	0	5	10	15	20		Combined	Linear	Quadratic
Moisture	33.82^a^	33.11^a^	32.27^b^	32.20^b^	31.96^b^	0.160	< 0.001	< 0.001	0.022
Crude protein	9.29^c^	9.70^b^	9.78^b^	9.89^b^	10.22^a^	0.069	< 0.001	< 0.001	0.617
Ether extract	0.65^e^	0.83^d^	1.04^c^	1.17^b^	1.29^a^	0.047	< 0.001	< 0.001	0.001
Crude fiber	0.66^e^	0.75^d^	0.92^c^	1.13^b^	1.31^a^	0.049	< 0.001	< 0.001	0.007
Ash	1.68^d^	1.75^d^	1.98^c^	2.27^b^	2.61^a^	0.071	< 0.001	< 0.001	< 0.001
Sugar	1.29^d^	1.37^d^	1.47^c^	1.91^b^	2.06^a^	0.063	< 0.001	< 0.001	< 0.001
Starch	48.05^a^	47.52^b^	46.81^c^	45.68^d^	45.12^e^	0.230	< 0.001	< 0.001	0.297

Abbreviation: SEM, standard error of mean.

^a,b,c,d,e^ Means within a row followed by the different superscripts differ significantly (*p* < 0.05).

In particular, quinoa addition led to significant enhancements (*p* < 0.001) in the mineral profile of lavash bread, including calcium, phosphorus, potassium, magnesium, iron, copper, zinc, and manganese, as shown in Table 6. The iron content in lavash bread with 20% quinoa flour was 2.75 times higher than that of the control sample. As iron is an essential trace element critical for human nutrition, and considering that iron deficiency remains a widespread public health concern globally, the enrichment of bread with iron through quinoa flour supplementation represents a promising nutritional strategy. In addition to iron, quinoa contributes other vital micronutrients, thereby improving the overall nutritional quality of the final product. These findings are consistent with those of Stikic et al. (2012), who demonstrated that adding 10%, 15%, and 20% quinoa flour to wheat flour significantly increased bread's protein, lipid, fiber, iron, manganese, and magnesium content relative to wheat flour alone.

**TABLE 6:** Mineral composition of lavash breads containing different percentages of quinoa flour.

Minerals	Quinoa flour (%)					SEM	*p* (significance)		
	0	5	10	15	20		Combined	Linear	Quadratic
Calcium (g/kg)	0.22^d^	0.26^c^	0.29^bc^	0.31^b^	0.37^a^	0.012	< 0.001	< 0.001	0.635
Phosphorus (g/kg)	0.95^a^	1.03^ab^	1.09^bc^	1.17^ab^	1.27^a^	0.119	< 0.001	< 0.001	0.451
Potassium (g/kg)	2.29^d^	2.66^c^	2.82^c^	3.41^b^	3.78^a^	0.127	< 0.001	< 0.001	0.154
Magnesium (g/kg)	0.31^d^	0.33^cd^	0.36^c^	0.41^b^	0.45^a^	0.012	< 0.001	< 0.001	0.133
Iron (mg/kg)	6.54^c^	8.41^c^	11.43^b^	13.99^b^	18.01^a^	0.962	< 0.001	< 0.001	0.105
Copper (mg/kg)	1.33^c^	1.40^b^	1.41^b^	1.41^b^	1.47^a^	0.011	< 0.001	< 0.001	0.438
Zinc (mg/kg)	8.69^d^	9.66^cd^	10.93^bc^	11.84^ab^	13.29^a^	0.393	< 0.001	< 0.001	0.640
Manganese (mg/kg)	6.74^d^	7.23^cd^	7.48^c^	8.18^b^	8.93^a^	0.185	< 0.001	< 0.001	0.108

Abbreviation: SEM, standard error of mean.

^a,b,c,d^ Means within a row followed by the different superscripts differ significantly (*p* < 0.05).

Amino acid composition analysis (Table 7) showed no statistically significant changes in levels of serine, proline, glycine, tyrosine, phenylalanine, histidine, methionine, cysteine, tryptophan, and cystine across the samples. However, quinoa supplementation led to significant increases (*p* < 0.05) in several essential amino acids, including aspartic acid, alanine, arginine, valine, leucine, and lysine. These results suggest that quinoa flour enhances the amino acid profile of lavash bread, particularly in terms of essential amino acids. Codina et al. (2016) similarly reported that the incorporation of 5%–10% quinoa flour improved bread's nutritional value and sensory characteristics. El‐Sohaimy et al. (2019) also noted that flatbread made from quinoa flour had higher contents of all essential amino acids, especially lysine, than that made from wheat flour, with the exception of cysteine and proline.

**TABLE 7:** Amino acid composition of lavash breads containing different percentages of quinoa flour.

Amino acids (%)	Quinoa flour (%)					SEM	*p* (significance)		
	0	5	10	15	20		Combined	Linear	Quadratic
Aspartic acid	0.235^c^	0.262^ab^	0.287^bc^	0.305^ab^	0.331^a^	0.008	< 0.001	< 0.001	0.751
Glutamic acid	1.610^ab^	1.612^a^	1.604^ab^	1.571^bc^	1.549^d^	0.007	0.001	< 0.001	0.050
Serine	0.300	0.304	0.311	0.312	0.313	0.002	0.148	0.020	0.393
Proline	0.871	0.880	0.879	0.876	0.866	0.003	0.615	0.560	0.150
Glycine	0.323	0.320	0.314	0.311	0.299	0.003	0.119	0.012	0.577
Alanine	0.390^c^	0.397^bc^	0.404^bc^	0.417^ab^	0.430^a^	0.004	0.001	< 0.001	0.419
Arginine	0.262^d^	0.279^cd^	0.296^bc^	0.312^ab^	0.329^a^	0.006	< 0.001	< 0.001	0.917
Threonine	0.218^b^	0.225^ab^	0.230^ab^	0.230^ab^	0.235^a^	0.002	0.052	0.004	0.556
Valine	0.387^c^	0.397^bc^	0.409^abc^	0.420^ab^	0.426^a^	0.004	0.003	< 0.001	0.758
Isoleucine	0.954^a^	0.924^a^	0.886^b^	0.870^b^	0.852^b^	0.009	< 0.001	< 0.001	0.157
Leucine	0.263^c^	0.289^c^	0.322^b^	0.340^ab^	0.367^a^	0.009	< 0.001	< 0.001	0.607
Tyrosine	0.212	0.221	0.214	0.220	0.227	0.002	0.308	0.088	0.686
Phenylalanine	1.055	1.040	1.023	0.994	0.834	0.029	0.072	0.013	0.162
Lysine	0.170^c^	0.181^bc^	0.188^bc^	0.199^ab^	0.211^a^	0.004	< 0.001	< 0.001	0.767
Histidine	0.172	0.176	0.178	0.180	0.194	0.003	0.093	0.012	0.336
Methionine	0.145	0.148	0.157	0.161	0.161	0.003	0.155	0.017	0.606
Cysteine	0.203	0.204	0.212	0.208	0.210	0.002	0.658	0.291	0.577
Tryptophan	0.331	0.332	0.339	0.342	0.340	0.002	0.439	0.090	0.674
Cystine	0.331	0.326	0.325	0.316	0.323	0.002	0.091	0.032	0.231

Abbreviation: SEM, standard error of mean.

^a,b,c,d^ Means within a row followed by the different superscripts differ significantly (*p* < 0.05).

The observed discrepancies between different studies may be attributed to variations in quinoa flour composition, processing methods, and interactions between quinoa and wheat flour components. Factors such as protein quality, starch structure, lipid composition, and fiber content may influence the bread's texture, color, and sensory traits. Moreover, differences in baking parameters, dough hydration, and ingredient quality likely contribute to variability in reported outcomes.

## 4 Conclusion

Quinoa seeds are rich in high biological‐value proteins, essential amino acids, minerals, vitamins, and bioactive components. Incorporating quinoa flour into wheat flour for lavash production enhances the bread's nutritional profile by increasing its protein, fat, minerals, and essential amino acid content. This approach enables the development of functional lavash with improved nutritional and sensory characteristics. The inclusion of quinoa flour at levels of 15%–20% significantly increased water absorption, extended dough development time, and raised the extensograph ratio. Therefore, quinoa flour can be effectively used at levels up to 20% in lavash wheat bread to improve both its nutritional and sensory qualities. Further studies could investigate the effects of quinoa flour at inclusion levels above 20%, as well as evaluate the antioxidant capacity and shelf life of quinoa‐enriched lavash bread.

## Author Contributions

**Mustafa Akturfan:** conceptualization (equal), formal analysis (equal), investigation (equal), methodology (equal), writing – original draft (equal). **Suzan Yalçın:** conceptualization (equal), formal analysis (equal), investigation (equal), methodology (equal), project administration (equal), writing – original draft (equal).

## Conflicts of Interest

The authors declare no conflicts of interest.

## Data Availability

The data that support the findings of this study are available from the corresponding author upon reasonable request.
